# Author Index for Volume 106

**DOI:** 10.1038/bjc.2012.247

**Published:** 2012-06-05

Aalfs, CM 2016

Aamdal, S 1728

Aapola, U 517

Aapro, M 1249

Abdelhady, S 1807

Abdel-Rahman, WM 517

Abel, GA 1021

Abel, GA 1068

Aboagye-Sarfo, P 1016

Abraham, J 32

Absher, D 307

Abson, C 262

Abu-Khalaf, M 1543

Aburatani, H 126

Acebes, JJ 1816

Ackland, SP 61

Adlard, J 2016

Adrover, E 1367

Agadjanian, H 333

Agaimy, A 719

Agarwal, R 1089

Agarwal, R 1107

Agelaki, S 1917

A'Hern, R 1760

Ahn, HS 733

Aitchison, M 279

Akiyama, F 1675

Akiyama, K 1214

Akslen, LA 1682

Aldridge, J 1618

Alessi, DR 1117

Alexandre, J 633

Alfaro, V 1379

Alferez, D 858

Ålgars, A 428

Algazi, AP 85

Alifano, M 1989

Ali, HR 1798

Allen, NE 1004, 1866

Allin, KH 199

Altiok, S 77

Altvater, B 1123

Al Zarouni, KMI 1166

Amiot, M 1660

Amsellem-Ouazana, D 1177

Anak, O 1475

Andäng, M 1807

Andersen, RS 1262

Anderson, ARA 174

Andersson, S 1297

André, F 1107

Andrews, SC 85

Androulakis, N 1917

Andrulis, IL 2016

Ankersmit, HJ 904

Annels, NE 496

Anthony, A 793

Antolín, I 1288

Antoniou, A 2016

Apostolaki, S 1917

Appleyard, MVCL 1117

Apte, SM 1967

Arao, T 727

Ardanaz, E 1004

Ardizzoni, A 658

Aretini, P 2016

Ariño, J 1816

Arita, T 740

Arizumi, T 1934

Armstrong, BK 638

Arnold, N 2016

A Roddam, A 210

Aroer, B 2016

Arrieta, Ó 1027

Arseneau, J 1460

Arús, C 1816

Ashcroft, MA 1638

Ashman, S 45

Ässämäki, R 517

Astsaturov, I 748

Atherly, AJ 1100

Auer, G 1297

Aukim-Hastie, C 157

Ayala, G 174

Azodi, M 1543

Azuma, K 763

Baader, K 719

Baba, H 182, 876

Baba, Y 182, 876

Baccarelli, A 585

Backen, A 508

Bäcklund, LM 1850

Baffet, G 685

Bagnulo, A 658

Bago-Horvath, Z 25, 440

Baird, MA 92

Baker, L 955

Baker, LCJ 1638

Baladandayuthapani, V 1374

Balázsi, G 1107

Baldini, E 658

Balko, JM 148

Ballester, M 39

Ball, G 1611

Balteskard, L 756

Banerjee, S 436

Bansal, N 1361

Baracos, VE 1583

Barbachano, Y 1718

Barbault, A 307

Barbiere, JM 1068

Barceló, A 1816

Baris, D 1891

Barjhoux, L 2016

Barnadas, A 1367

Barone, C 658

Barrett-Lee, P 32

Barrett, S 1728

Barriuso, J 1246

Barriuso, J 1728

Barry, M 805

Barton, S 1760

Bartsch, R 25, 440

Basanta, D 174

Basketter, V 157

Basso, U 1247

Bastiaannet, E 1564

Battegay, M 447

Bauer, E-M 1551

Beale, GS 1386

Bearz, A 1899

Becker, S 1004, 1297, 1866

Beeken, RJ 1907

Beer, H 45, 1753

Beesley, J 2016

Begum, S 414

Beijnen, JH 1598

Bekaii-Saab, T 1583

Bellone, S 1543

Belotti, M 2016

Belvederesi, L 799

Benítez, J 2016

Benner, A 1239

Bentley, RC 916

Beral, V 210

Bergamaschi, D 1446

Berger, MS 839

Berger, R 189

Berghoff, A 25, 440

Bergkvist, L 592

Berkhof, J 817, 975

Bernasconi, E 447

Berndt, SI 608

Berney, DM 1095

Bernhard, J 1618

Berretta, M 424, 1899

Berrino, L 1648

Berry, G 1016

Berry, MP 638

Betof, AS 916

Beyene, J 1626

Beyer, M 1262

Bezu, C 39

Bhadkamkar, NA 1374

Bhattarai, BN 962

Bhopal, RS 1361

Bianchini, G 1107

Bianconi, M 799

Bible, KC 314

Bicaku, E 1967

Bickers, B 157

Bidstrup, PE 1560

Bieche, I 1177

Biggs, P 262

Birkeland, E 1682

Bittoni, A 799

Bitzer, M 1033

Bjerkehagen, B 297

Black, EP 148

Blackhall, F 508

Blackman, CF 241

Blackwell, KL 916

Blakely, J 839

Bland, MJ 1753

Blank, MM 596

Blann, AD 1466

Blighe, K 375

Bliss, JM 1062

Blok, MJ 2016

Blomqvist, C 1076

Blomqvist, P 1850

Blows, FM 1798

Board, R 508

Bodin, I 1297

Bodin, T 1083

Bodman-Smith, M 896

Boeck, S 1033

Boeing, H 1004, 1866

Boessenkool-Pape, JL 2016

Boffetta, P 575

Böhling, T 1076

Boix, O 1722

Bojesen, SE 199

Boku, N 1268

Bolognesi, C 780

Boluda, S 1816

Bomalaski, J 324, 1481

Bonanni, B 2016

Boni, C 658

Boni, L 658

Böning, L 274

Bonin, S 166

Bonneterre, J 673

Bono, Y 1205

Borg, M 61

Bornfeld, N 1171

Borre, M 366

Bortolomai, I 1543

Bosch-Barrera, J 1406

Boss, DS 1598

Bossou, AR 314

Bouchardy, C 447

Boukovinas, I 453

Boult, JKR 1638, 1960

Bourgkard, E 1346

Bouscary, D 1660

Boutron-Ruault, MC 1004, 1866

Boutry-Kryza, N 2016

Bove, B 748, 2016

Boven, E 1728

Bowser, R 2004

Boykin, C 1248

Bozionelou, V 1917

Bracarda, S 1475

Bradley, MC 233

Brain, E 673

Brait, M 414, 1314

Bramsen, JB 366

Bray, SE 1117

Breitbart, EW 970

Brenner, H 569, 1424, 1875

Brenn, T 553

Brewster, DH 1361

Brezovich, I 307

Briasoulis, E 1446

Briel, M 1626

Bright, JJ 1702

Britson, J 148

Broadwater, G 916

Brockhoff, G 719

Brown, A 425

Brown, C 61, 633

Brown, CH 1068

Brown, ERS 553

Brown, J 375

Brown, M 1689

Brown, NJ 1153

Bruland, O 756

Brunet, J 1406

Bruzzi, P 780

Bryant, JL 148

Bryant, RJ 768

Büchert, M 1722

Buchwald, C 1526

Buckholz, J 996

Buechler, P 1033

Bueno-de-Mesquita, HB 1004, 1866

Buettner, M 1980

Bulk, S 975

Buonerba, C 617

Burchill, SA 1224

Burger, EA 1571

Burke II, J 70

Burke, W 1728

Burnell, M 1910

Burnley, C 1753

Bushmakin, AG 646

Butler-Manuel, S 482

Butler, S 1234

Butow, P 1053

Büttner, AC 1980

Butt, S 389

Buza, N 1543

Bydder, S 61

Byrd, PJ 262

Byrnes, G 575

Byrski, T 2016

Cafferata, MA 658

Cagnard, N 1989

Cai, H-M 1735

Cai, KQ 748

Cajal, TR 2016

Caldas, C 1798

Caley, A 32

Califano, J 1314

Calmy, A 447

Calvo, E 1475

Cameron, S 107

Camidge, DR 1100

Campbell, C 1, 1262

Campbell, S 358

Candiota, AP 1816

Cantor, KP 1891

Cantwell, MM 233

Capasso, A 1648

Capdevila, J 1379

Cappelleri, JC 646

Caraglia, M 1648

Cardinale, F 780

Cardoso, F 1618

Carducci, MA 77

Carlson, J 389

Carlsson, E 517

Carmichael, J 468

Carney, EF 262

Caroti, C 658

Carpén, O 428

Carpenter, LM 1556

Carroll, S 61

Casado-Zapico, S 1288

Cascinu, S 799

Cassapa, C 1866

Cassidy, J 793

Castañon, A 45

Castells, X 1816

Castro, E 1697

Catto, JWF 768

Caux-Moncoutier, V 2016

Cavassini, M 447

Ceelen, W 1926

Celano, P 70

Cella, D 646

Chalchal, H 633

Chalmers, AJ 18

Chang, C-J 243

Chang, CM 206

Chang, C-Y 475

Chang, H-M 1039, 1591

Chang, J 116

Chang, KC 693

Chang, K-Y 475

Chang, T-Y 475

Chanthirakumar, C 896

Chao, Y-S 475

Charbonneau, C 646

Chauffert, B 460

Chau, I 1718

Cheah, BC 1053

Chen, CJ 206

Chen, C-JJ 1134

Chen, C-P 475

Chen, C-T 475

Chen, C-W 475

Chen, D-K 1735

Chen, D-T 1967

Chenevix-Trench, G 2016

Cheng, JTY 1486

Chen, JY 206

Chen, N 1967

Chen, S 1512

Chen, X 1320, 2016

Chen, Y-H 1248

Cheon, JH 53

Cherala, S 1891

Chéreau, E 39

Chiappori, AA 839

Chilcott, J 805

Ching, L-M 1134

Chiocchia, G 1989

Chivers, P 482

Chivukula, M 2004

Chiyomaru, T 405

Cho, EY 923

Choi, D 1833

Choi, I 1833

Choi, JY 923

Choi, SH 53

Choi, Y-L 923

Chong, M-h 1605

Chon, HS 1967

Chow, W-H 585

Christensen, O 1722

Christophyllakis, C 453

Chuang, S-C 1866

Chu, ESH 1486

Chung, HK 1833

Chung, Y-L 1638

Chu, QS 839

Chu, W 693

Ciardiello, F 1648

Cimino, Y 1989

Ciuffreda, LP 1648

Clack, G 508

Claes, K 1460

Clancy, T 775

Clarke, A 775

Clarke, N 1689

Clavel-Chapelon, F 1004, 1866

Clayton, R 775

Clearly, S 955

Clément-Duchêne, C 1346

Clements, K 1611

Clifford, GM 447

Coates, AS 1618

Coates, PJ 1117

Cocco, E 1543

Coebergh, JW 1564

Coeffic, D 633

Coffin, RS 496

Cohen, SJ 748

Colantuoni, E 1314

Cole, T 2016

Coley, HM 482

Colin, P 1083

Colt, JS 1891

Combe, M 460

Comber, H 805

Comino, A 397

Concannon, CG 1499

Concin, N 1551

Conlon, S 1499

Constantinescu, SN 1249

Cook, J 2016

Coombes, RC 375, 1062, 1790

Copalu, W 1598

Corcos, L 685

Corona-Cruz, JF 1027

Coronado, C 1379

Cortes-Funes, H 1379

Costa, FP 307

Costantino, S 1648

Costes, A 1989

Cottura, E 673

Couch, F 2016

Coulton, G 896

Coupe, VMH 975

Coupier, I 2016

Courouble, N 1346

Coutant, C 39

Cox, RF 525

Cozzi, P 638

Cree, IA 157

Cristiano, L 166

Crivellari, D 1247

Crocchiolo, R 966

Crook, T 397, 482, 1446

Cross, AJ 608, 1891

Crouzet, S 1083

Crowe, FL 1004

Crowley, LC 711

Crowther, MJ 1854

Crumley, ABC 702

Cubitt, CL 1967

Cufí, S 1406

Cummings, J 1766

Cummins, MM 61

Cummins, RJ 1499

Cunningham, D 1718

Curtis, LB 629

Cuschieri, K 358

Cussenot, O 1083

Cuvelier, CA 141

Cuzick, J 1095

Cybulski, C 2016

Dadaev, T 1697

Daenen, LGM 1901

Dahler, A 107

Dahl, O 756

D'Aiuto, E 1648

Dalenc, F 1579

Dalenc, F 673

Dalgleish, AG 896

Dalton, SO 988, 1560

Damery, S 1431

Damotte, D 1989

D'Andrea, RJ 1772

Dang, TP 1953

Danielidis, V 846

Dapas, B 166

Daraï, E 39

Daud, AI 85

David Batty, G 1842

Davidson, R 2016

Davies, BR 858

Davies, E 651

Davies, JE 14

Dawnay, A 1910

Dawson, CW 1980

Dawson, S-J 1798

Dean, E 468

de Bono, JS 1379

Debre, B 1177

De Bruyne, S 1926

Decaestecker, C 141

DeConti, RC 85

de Craen, AJM 1564

Deen, S 1306

de Gast, B 284

de Hullu, JA 269

Deissler, H 2016

Dekkers, OM 1564

de Klerk, NH 1016

De Leeneer, K 1460

de Llorens, R 1406

de Long, LM 107

De Lorenzi, M 1648

Del Prete, M 799

Delroeux, D 460

Del Vecchio, CA 883

de Martino, M 904

Demetter, P 141

Dennis, JL 896

de Nully Brown, P 988

De Palma, R 1648

De Paoli, P 966

De Placido, S 617

de Rooi, JJ 538

Descamps, G 1660

Descazeaud, A 1083

Desfrere, L 1807

deSouza, NM 619

Detjen, KM 348

Devilee, P 2016

DeVries, C 25, 440

de Vries, EGE 1728

Dewdney, A 1718

Dewhirst, MW 916

Dicker, P 1499

Dickinson, PD 1464

Di Costanzo, F 658

Dieckmann, K 25, 440

Diehl, SR 206

Diéras, V 673, 854

Dierkes, C 1123

Di Fiore, F 426

Dilis, V 1004

Dillner, J 389

Di Lorenzo, G 617

Dinjens, W 791

Dippold, W 1239

Dirksen, U 1123

Dive, C 508, 1766

Dmoszynska, A 546

Doble, A 436

Dodds, M 1779

Doglioni, C 966

Dognini, G 966

Doherty, V 553

Doi, T 666

Doki, Y 947, 1415

Dolcetti, R 966

Domchek, SM 2016

Dommett, RM 982

Done, SJ 1506

Dong, C 889

Donnelly, P 1

Dorronsoro, M 1004, 1866

Dotan, E 748

Dote, N 666

Doughty, J 1453

Doughty, JC 383

Dowsett, M 1760

Dowswell, G 1431

Doyle, LA 1583

Dralle, H 562

Drogan, D 1004

du Bois, A 629

Dubsky, P 25, 440

Duell, EJ 1004, 1866

Dunbar, PR 1134

Duncan, M 1187

Du, Q 1605

Durocher, F 2016

Durrant, LG 1306

Dutta, B 1107

Dutta, S 702

Dyrskjøt L 366

Easton, D 1697

Easton, DF 2016

Eberhard, J 931

Eberhard, K 1826

Eberle, A 1875

Eblis, SD 2016

Eccles, D 1234, 2016

Eccles, S 883

Eckhardt, J 1980

Edwards, J 383, 702, 1187, 1453

Edwards, RP 2004

Eeles, R 1697, 2016

Egger, M 447

Egle, D 189

Ehret, C 826

Ehrnrooth, E 793

Eilers, PHC 538

Eisemann, N 970

Eiter, H 440

Elgart, G 1481

El-Khamisy, SF 18

Elliott, J 1

Elsberger, B 383, 1453

El-Serag, H 1011

Elvin, P 1960

Elzinga, B 711

Emeribe, U 1728

Emery, J 1262

Emrich, K 1875

Endlicher, E 1033

Endo-Munoz, L 107

Endo, T 1268

Eng, C 1374

Engelsen, IB 1682

Engström, U 1297

Enokida, H 405

Erickson, RL 1331

Erlich, RB 107

Ernst, B 826

Errington-Mais, F 92

Escudier, B 1475

Escuin, D 1367

Esmarck, B 793

Essapen, S 883

Essers, MAG 1742

Ess, S 447

Estrada-Lobato, E 1027

Ethier, MC 1626

Ettl, T 719

Evans, A 1446

Evans, DG 1234, 2016

Evans, DGR 775

Evans, TRJ 1598

Exley, A 262

Exon, D 955

Eyfjörd, JE 389

Ezan, F 685

Falci, C 1247

Fall, K 217

Fallowfield, LJ 1062

Faloppi, L 799

Fan, X-E 1248

Fan, X-J 1735

Faratian, D 1779

Farra, R 166

Farrell, GC 1486

Faulkner, K 1753

Fedirko, V 1004, 1866

Fehr, J 447

Feliu, J 1246

Felix, AS 2004

Fentiman, IS 1467

Ferdinande, L 141, 1926

Ferguson, T 1760

Fernández-Teruel, C 1379

Feron, J-G 39

Ferreri, AJM 966

Ferrero, A 633

Ferrone, S 939

Ferrucci, LM 608

Feun, LG 1481

Févotte, J 1346

Fiander, A 45

Fiduccia, P 1247

Fiegl, H 189

Fielder, H 45

Figlin, RA 1475, 1587

Filleron, T 1579

Fineberg, E 2016

Finn, RS 6

Fisher, G 1095

Fisher, RA 1089

Fishman, MN 174

Fitzal, F 25, 440

Fitzharris, B 633

Flanagan, JM 248

Flegg, KM 2010

Fleming, C 1446

Fleming, S 1117

Fleuren, GJ 1520

Fleuter, C 1395

Flugelman, A 2016

Fluge, Ø 756

Fodde, R 1564

Follet, J 685

Fong, PC 1379

Formentini, A 1239

Försti, A 389

Fortuzzi, S 2016

Fosså, SD 297

Foster, CS 1095

Foulkes, WD 1460

Fourkala, EO 1910

Fowler, DW 896

Fowler, S 638

Franceschi, S 222, 447

Franklin, P 1016

Frebourg, T 426

Frede, A 1772

Fredericksen, Z 2016

Frederiksen, BL 988

Frederiksen, K 1560

French, PJ 538

Freyer, G 1728

Fricker, J-P 2016

Friedlander, M 1053

Friess, H 133, 792

Friis-Hansen, L 1526

Friis, S 1353

Frisk, G 1850

Fristrup, N 366

Frolov, A 148

Frost, A 1722

Frost, D 2016

Fuchs, CS 1335

Führer, D 562

Fujie, M 1214

Fujii, M 1196

Fujimoto, M 666

Fujisawa, M 1945

Fujiwara, H 740

Fujiwara, Y 947, 1415

Fullarton, GM 702

Fulp, W 1967

Fumoleau, P 673

Fu, Q 1512

Fuse, M 405

Fuse, N 666

Fu, X-H 1735

Fyffe, G 1187

Gaber, A 931

Gaffney, EA 1280

Galaal, K 1753

Gale, CR 1842

Galizia, E 799

Gallardo, A 1367

Gao, S 366

Gao, Y 585

Gao, Y-T 585

Garbis, SD 157

García-Santos, G 1288

Garnæs, E 1526

Garrett, CR 1374

Garrone, O 397

Gasparrini, S 1543

Gatenby, RA 1280

Geboes, K 1926

Gebski, VJ 61

Geissler, M 1033

Gelber, RD 1618

Geller, AC 970

Gemma, A 867

Gemoll, T 1297

Gentry-Maharaj, A 1910

Georgoulias, V 1917

Georgoulias, V 453

Germano, D 658

Ghatage, P 70

Gheit, T 222

Ghia, P 966

Ghosh, S 1583

Giacalone, A 424

Giampieri, R 799

Giannopoulos, K 546

Giannopoulos, T 482

Giansante, C 166

Giatromanolaki, A 846

Gilbert, DC 18

Gilbert, M 2016

Giles, R 284

Gillam, MC 116

Gill, G 1045

Gillies, RJ 1280

Gillis, AJM 791

Gilmour, LD 1960

Giner, D 1367

Giovannucci, EL 1335

Girard, L 116

Giri, P 962

Giurescu, M 70

Glauche, I 1742

Glazer, S 678

Gleave, ME 1945

Glendon, G 2016

Glen, H 1598

Glud, M 1526

Gnanapragasam, VJ 436

Gnant, M 25, 440

Godwin, A 2016

Godwin, AK 748

Goebel, G 189

Goh, C 1697

Gohda, K 133, 792

Going, JJ 383, 702, 2010

Gojis, O 397

Goldgar, D 1697

Goldhirsch, A 1618

Goldstein, AM 206

Goldstein, D 61

Golemis, EA 748

Gomez-Bougie, P 1660

Gondos, A 1875

Gonzalez-Bosquet, J 1967

Gonzalez, M 1346

Goodege-Kunwar, P 468

Gorter, A 1520

Gosau, M 719

Gosheger, G 1123

Goto, M 666

Goto, Y 939

Gourley, C 358

Govindasami, K 1697

Govi, S 966

Graf, D 562

Granath, F 1860

Grassi, G 166

Grassi, M 166

Graubard, BI 1331

Gravendeel, LAM 538

Gravett, AM 896

Green, A 1660

Greenberg, DC 1068

Green, J 210

Green, JA 1581

Grefte, JMM 269

Greinert, R 970

Grimm, C 1551

Groenewegen, G 284

Grønbæk, H 1004

Grønbæk, M 1560

Gronwald, J 2016

Groot-Obbink, K 1772

Grossi, F 658

Grote, VA 1004, 1866

Grünwald, V 1475

Grzybowska, E 389

Guardiola, E 460

Guminski, A 107

Gunter, MJ 227

Guo, X 333

Gurrea, AB 1866

Gurung, N 962

Gustafson, P 1076

Gutin, A 1095

Gutknecht, M 562

Guttery, DS 375

Guy, M 1697

Haagensen, EJ 1386

Haanen, J 284

Habermann, J 1297

Hagiwara, M 290

Hagiwara, S 1997

Haitel, A 904

Hakam, A 1967

Hakulinen, T 569, 1846

Halachmi, S 414

Halbert, G 1187

Halkjær, J 1866

Hall, A 1697

Hallas, J 1353

Halliday, J 1960

Hall, KS 297

Hamaguchi, T 727

Hamann, U 2016

Hamano, R 1415

Hamdy, FC 768

Hamel, N 1460

Hamilton, W 982, 1262, 1940

Han, C 1466

Hanna, W 1160

Hansen, LT 793

Hansen, TB 366

Hanson, H 1234

Harada, K 939

Hardee, ME 916

Harder, J 1033

Hardes, J 1123

Harding, V 1089

Hardwick, JCH 1495

Harlid, S 389

Harmey, JH 525

Harrington KJ 496

Harris, AL 1466

Harris, D 1379

Harris, HR 592

Harris, LN 916

Harrison, DJ 1779

Harris, S 157

Hart, C 1689

Harter, P 629

Hartmann, A 1980

Hartog, V 1728

Hart, R 1053

Harvey, J 61

Harvey, R 1089

Harvey, V 1618

Harwood, C 1446

Hasegawa, Y 1196

Hasmann, M 1779

Hassabo, HM 1374

Hassan, MM 1374

Hastings, BT 1021

Hatashita, E 763

Hatzimichael, E 1446

Hauff, P 348

Haug, U 1424

Hautaniemi, S 517

Hava, NL 375

Hawinkels, LJAC 1495

Hayashi, N 876

Hayes, RB 608

Häyry, V 517

Hayward, SW 174

Hazar-Rethinam, M 107

Healy, S 2016

Heaton, SP 858

Hector, S 1499

Heideman, DAM 975

Heier, A 858

Heinemann, V 1033

Heinig, R 1722

Hei, TK 1512

Helle, M 517

Hellman, K 1297

Hellman, U 1297

Hellström, A-C 1297

Helmerhorst, TJM 817

He, M 889

Hemminki, K 389

Henare, K 1134

Herbst, K 2016

Herings, RMC 1564

Hernández-Pedro, N 1027

Hernandez-Santana, A 525

Herrera, F 1288

Herrstedt, J 1353

Heyd, B 460

Hickey, M 1053

Hida, K 1214

Hida, Y 1214

Hildesheim, A 206

Hills, M 1760

Hilmy, M 1187

Hilvo, M 99

Hinchliffe, SR 1854

Hiom, S 1262

Hirakawa, K 1535, 1668

Hirano, K 1934

Hirano, S 1214

Hirashima, K 876

Hirashima, S 740

Hite, KM 1395

Hiyoshi, H 1807

Hiyoshi, Y 182

Hobbs, FDR 1431

Hoefler, G 1826

Hoetzenecker, K 904

Hofheinz, R 1033

Hofstädter, F 826

Hofstetter, G 1551

Holder, R 1431

Holleczek, B 1875

Hollenbeck, A 596

Holmes, S 157

Holt, SV 858

Hommes, DW 1495

Hong, Y-K 1833

Hoogland, AM 791

Hoque, MO 414

Horgan, PG 702, 1187, 2010

Horii, R 1675

Horn, K 1742

Horn, M 1742

Hortobagyi, GN 1107

Horton, JK 916

Horvath, A 496

Horvath, L 61

Hosoya, T 1148

Hossain, I 638

Hotchkiss, R 693

Hotfilder, M 1123

Hou, L 585

Houlston, R 1234

Housley, SL 279

Houthuijzen, JM 1901

Howard, K 1045

Howell, I 1234

Hruby, G 61

Hsieh, S-Y 475

Hsu, JT-A 475

Hsu, WL 206

Huang, W-Y 608

Huang, X 1587

Huang, Y 1735

Huang, Y-L 475

Hubbard, G 1

Huerta, J-M 1004

Hughes, A 508

Hughes, CM 233

Hughes, D 1234

Hughes, TP 1772

Hulbert-Williams, N 1

Hung, M-C 243

Hupertan, V 1083

Hur, H 53

Hus, I 546

Husslein, H 1551

Hutson, TE 1475

Hutson, TE 1587

Huzarski, T 2016

Hwang, J 85

Hyötyläinen, T 99

Ichikawa, D 740

Ichikawa, T 405

Ide, H 290

Ihorst, G 1033

Ijichi, K 1196

Ikejiri, K 1268

Ikezawa, Y 1953

Iljin, K 99

Imai, S 1148

Imamura, Y 876

Immervoll, H 756

Im, Y-H 923

Ingham, S 775

Inman, GJ 1446

Inoue, M 1205

Inoue, T 1997

Irving, S 436

Isaacs, C 2016

Isambert, N 673

Isayama, H 1934

Isham, CR 314

Ishihara, H 133

Ishihara, H 792

Ishii, H 947

Ismail, T 1431

Itoh, S 1976

Ito, Y 1934

Ivarsson, MIL 389

Iwase, T 1675

Iwatsuki, M 182

Iwauchi, T 1668

Iyama, K-I 876

Izatt, L 262

Jacob, J 375

Jacobs, C 2016

Jacobs, I 1910

Jacobs, V 1960

Jakubowska, A 2016

James, J 1431

Jamin, Y 1638

Jamin, Y 1960

Janne, PA 763

Jansen, L 569

Jansen, L 1875

Janssen, K-P 133

Janssen, K-P 792

Jasani, B 32

Jeffers, M 1722

Jehn, CF 274

Jelkmann, W 1249

Jenab, M 1004

Jenab, M 1866

Jensen, TI 366

Jenster, G 791

Jeurnink, S 1004

Jiaang, W-T 475

Ji, B-T 585

Jimenez, M 673

Jinno, F 666

Jirström, K 931

Jitlal, M 1153

Joffe, S 1021

Johannsson, OTh 2016

Johansen, C 1560

Johansen, D 1004

Johansen, D 1866

Johansen, JS 199

Johnsen, JI 1807

Johnson, A 1891

Johnston, PG 1499

Jo, J-C 1591

Jones, A 189

Jones, EA 775

Jones, R 1728

Jordanova, ES 1520

Jørgensen, TL 1353

Jost, A 1274

Judson, PL 1967

Juergens, H 1123

Jukkola-Vuorinen, A 344

Julià-Sapé, M 1816

Jullien, V 460

Jundt, G 447

Jungbluth, AA 1481

Jungbluth, AA 324

Jung, J 1506

Junor, E 358

Kaaks, R 1004

Kaaks, R 1866

Kabat, GC 227

Kaga, K 1214

Kailayangiri, S 1123

Kakolyris, S 453

Kalbacher, E 460

Kalbakis, K 1917

Kalland, KH 1682

Kallioniemi, O 99

Kameoka, S 1268

Kaminska, E 896

Kaminska, W 546

Kamohara, H 182

Kanakasabai, S 1702

Kang, KN 733

Kang, S 1314

Kang, WK 1469

Kang, Y-K 1039

Kang, Y-K 1591

Kantala, J 2016

Kantelip, J-P 460

Karagas, MR 1891

Karamitopoulou, E 1713

Karashima, R-I 876

Karenko, L 517

Karger, S 562

Karihtala, P 344

Karim, R 1520

Karlan, BY 333

Karlsson, P 2016

Katalinic, A 1875

Katalinic, A 970

Katki, HA 608

Kato, K 727

Kauppila, S 344

Kawaguchi, T 740

Kawakami, K 405

Kawamoto, T 1214

Kay, C 1779

Kaye, S 1728

Kaye, SB 619

Kay, EW 1499

Kearins, O 1611

Kee, BK 1374

Keesen, M 1598

Keiser, O 447

Kelly, MP 324

Kelly, P 955

Kentepozidis, N 453

Kenter, G 817

Kernohan, NM 1117

Kerr, G 358

Ketola, K 99

Keun, H 909

Khan, G 1166

Khan, OA 1466

Khatchadourian, V 1833

Khaw, K-T 1004

Khaw, K-T 1866

Khelwatty, SA 883

Kherrouche, Z 107

Khoja, L 508

Kieler, H 1860

Kikuchi, E 290

Kikuchi, E 1953

Kikuchi, J 1953

Kilburn, LS 1062

Kim, CW 733

Kim, D 1475

Kim, HG 53

Kim, H-S 1591

Kim, JJ 1571

Kim, M 1591

Kim, MY 227

Kim, NK 53

Kim, NY 1833

Kim, SJ 916

Kim, ST 1587

Kim, TW 1039

Kim, T-W 1591

Kimura, K 1675

Kim, Y 733

Kim, YJ 923

King, MT 638

Kiniwa, Y 939

Kinoshita, K 182

Kinugasa, Y 1268

Kirichek, O 210

Kirk, RS 883

Kitamura, K 867

Kitayama, K 1214

Kiyono T 1205

Kjems, J 366

Klatte, T 904

Klinkhammer-Schalke, M 826

Kloeppel, G 1033

Kloor, M 1239

Kluijt, I 2016

Knauer, M 440

Knuutila, S 517

Koch, WM 1314

Kocken, M 817

Kogner, P 1807

Kogure, H 1934

Koike, K 1934

Koizumi, F 727

Kojima, S 405

Kok, CH 1772

Koller, M 826

Komatsu, S 740

Kondo, E 1196

Kondoh, M 1214

Konishi, H 740

Kopacek, A 1395

Kornmann, M 1239

Korol, D 447

Kosaka, T 290

Kotake, K 1268

Kote-Jarai, Z 1697

Koukourakis, MI 846

Kouroussis, C 453

Kouzeli, I 358

Kowalski, J 414

Krakstad, C 1682

Kramar, A 1579

Krause, K 562

Krebs, M 508

Krell, J 1790

Kristensen, GB 1728

Kristiansen, IS 1571

Kristjansen, PEG 793

Kristoffersson, U 2016

Krogh, V 1866

Krohn, K 517

Kroll, ME 1556

Kröning, H 274

Kubben, FJGM 1495

Kubo, N 1668

Kudo, M 1997

Kuenen, B 1728

Kühnel, T 719

Kuhn, K 1424

Kuo, MT 1481

Kuppen, PJK 133

Kuppen, PJK 792

Kurashige, J 182

Kuroda, M 1976

Kurokawa, Y 947

Kurokawa, Y 1415

Kuslich, C 768

Kuster, N 307

Kususda, Y 1945

Kuwata, K 763

Kuze, Y 666

Kuzumaki, N 1148

Kwon, MJ 923

Kwon, MJ 923

Kwon, S 333

Kyle, S 1386

Kyo, S 1205

Läärä, E 1846

Labianca, R 658

LaBonte, MJ 1833

Ladner, RD 1833

Lagergren, J 1011

Lagergren, J 1342

Lagiou, P 1004

Lajer, CB 1526

Lajer, H 1526

Lalloo, F 775

Lalloo, F 2016

Lal, R 1379

Lamarca, A 1246

Lambert, PC 1854

Lamb, GWA 279

Lamont, D 955

Lancaster, JM 1967

Lanchbury, JS 1095

Landmeier, S 1123

Lane, D 227

Langdon, SP 1779

Langers, AMJ 1495

Langridge, C 1062

Lang, S 189

Larkin, SET 157

Larsson, SC 603

Lassen, U 678

Last, JI 262

Lattanzio, L 397

Lattanzio, L 1446

Lau, M 1689

Laurent, S 1926

La Vecchia, C 222

Lawrence, GM 1611

Lax, S 1826

Lazar, V 1107

Lederman, RI 1021

Lee, C 1039

Lee, CK 1045

Lee, HJ 1039

Lee, HJ 1591

Lee, H-J 733

Lee, J 1469

Lee, J 1469

Lee, JE 1881

Lee, J-L 1039

Lee, J-L 1591

Lee, KY 53

Lee, S 1446

Lee, SM 1153

Leeson, S 1753

Lee, T 1668

Lee, YS 1833

Legius, E 1460

Le Gouill, S 1660

Lehrnbecher, T 1626

Leigh, I 1446

Leipold, H 1551

Lejbkowicz, F 2016

Le Jossic-Corcos, C 685

Lemmens, V 1564

Lenardon, O 222

Lenner, P 389

Lenz, H-J 1833

Leongamornlert, D 1697

Le Pessot, F 426

Lerma, E 1367

Le Tourneau, C 854

Leuchte, K 1123

Leunen, K 1460

Levi, F 447

Leyland-Jones, B 1249

Libbrecht, L 1926

Lidbrink, E 1850

Lidereau, R 1177

Liefers, GJ 1564

Li, G 1486

Li, H-L 585

Li, J 1512

Liljegren, A 2016

Lim, AKP 1089

Lim, HY 1469

Lindam, A 1342

Lindkvist, B 1004

Lindkvist, B 1866

Lindor, N 2016

Lindquist, KE 931

Ling, L-j 1605

Link, K-H 1239

Linkov, F 2004

Lintunen, M 428

Lin, W-H 475

Lise, M 447

Liu, MY 206

Liu, Q 1735

Liu, WM 896

Liu, X-a 1605

Lo, CM 1486

Logié, A 858

Lohmann, DR 1171

Lokiec, F 673

Loman, N 2016

Lomnytska, MI 1297

Long, JA 1083

Long, JS 1453

Lo Nigro, C 397

Lönn, S 1860

Looijenga, LHJ 414

Looijenga, LHJ 791

López-Martin, JA 1379

López-Rodríguez, V 1027

Lord, SJ 1045

Lorenz, W 826

Loretelli, C 799

Loud, JT 2016

Lou, PJ 206

Louwers, JA 817

Lovell, DP 482

Lowenstein, CL 1021

Loyo, M 414

Loyo, M 1314

Lubinski, J 2016

Ludvigsson, JF 217

Luecke, A 1123

Luftig, MA 429

Lüftner, D 274

Lugli, A 1713

Lundgren, C 1297

Lundgren, TK 1807

Lundin, J 1076

Lundin, M 1076

Luo, C 1512

Luo, J 227

Luo, Y 1790

Lu, Q 1248

Lyratzopoulos, G 1068

Maak, M 133

Maak, M 792

Ma, C 1506

Macartney, J 1611

Maccaroni, E 799

Macedo, EO 1027

Mach, RH 693

Mack, MG 1274

Mader, RM 25

Mader, RM 440

Madhuri, K 482

Mäenpää, J 633

Mahier-Aït Oukhatar, C 673

Mahmud, N 1697

Mahoney, MC 996

Maida, Y 1205

Maïga, S 1660

Maini, PK 1280

Mai, PL 2016

Maishi, N 1214

Ma, J 1335

Majek, O 1875

Major, JM 206

Majós, C 1816

Makrantonakis, P 453

Maldonado, L 414

Mallon, E 383

Mallon, E 1453

Manchanda, R 1910

Manjer, J 389

Man, K 1486

Marchion, DC 1967

Marini, A 1481

Marini, G 658

Marsden, G 768

Martens, J 791

Marth, C 1551

Martin, A 1045

Martin-Castillo, B 1406

Martinelli, E 1648

Martinet, Y 1346

Martínez-Barrera, L 1027

Martinez, ED 116

Martin-Hirsch, P 1753

Martin, NK 1280

Martin, RM 982

Martins, Y 1021

Martin, V 1288

Maseki, S 1196

Massuger, LFAG 269

Masuda, T 290

Masumura, K 1675

Matano, E 658

Mathieu, A 141

Matin, R 1446

Matsubara, S 1934

Matsubara, Y 1268

Matsuda, K 1214

Matsumoto, K 290

Matsumoto, K 727

Matsuoka, T 1535

Matsushima, T 133

Matsushima, T 792

Matthijs, G 1460

Mattiello, A 1004

Mattsson, F 1011

Mavroudis, D 1917

Ma, X-J 1790

Maxwell, RJ 1386

May, K 1506

Mazoyer, S 2016

Mazur, A 1598

McArdle, PA 1187

McCalmont, T 85

McCormack, V 575

McGlynn, KA 1331

McGuffog, L 2016

McHugh, A 1446

McKenna, SL 711

McMahon, G 525

McMeekin, S 70

McMillan, DC 279

McMillan, DC 383

McMillan, DC 702

McMillan, DC 2010

McMillan, DGG 92

Medina, LA 1027

Mefti-Lacheraf, F 673

Meijer, CJLM 817

Meijer, CJLM 975

Meijers-Heijboer, HEJ 2016

Meindl, A 2016

Meiser, B 1053

Mei, T 1512

Melcher, AA 92

Melcher, AA 496

Melin, B 2016

Melton, DW 553

Meltzer, J 1123

Menasce, L 508

Mencoboni, M 658

Menendez, JA 1406

Menkiszak, J 2016

Menon, U 1910

Merchan, JR 314

Merlano, M 397

Merlano, M 1446

Merler, E 1016

Meropol, NJ 748

Mertens, J 1926

Mesher, D 45

Mesher, D 1095

Messina, JL 85

Metcalf, S 496

Michaelson, MD 646

Michaelson, MD 1587

Michaud, DS 1004

Michaud, DS 1866

Michel, S 1239

Michils, G 1460

Middleton, MR 468

Middleton, MR 1466

Middleton, MR 1766

Midorikawa, Y 126

Miller, C 333

Mills, GB 1107

Mimori, K 182

Min, BS 53

Minegishi, Y 867

Mints, M 1297

Mistou, S 1989

Mitchell, C 1556

Mitrica, I 629

Miyagaki, H 947

Miyajima, A 290

Miyakawa, A 1807

Miyake, H 1945

Miyanaga, A 867

Miyata, H 947

Miyata, H 1415

Mizugaki, H 1953

Mizumoto, Y 1205

Mizutani, H 867

Mladkova, N 1446

Mochizuki, H 1268

Mochizuki, I 1268

Modi, B 116

Modjtahedi, H 883

Moehler, M 1033

Moezi, MM 839

Moffat, F 1481

Mohammed, Z 702

Mohammed, Z 2010

Mohammed, ZMA 383

Moles Lopez, X 141

Molina-Montes, E 1004

Molino, AM 1247

Møller, H 1095

Monfardini, S 1247

Montange, D 460

Monteverde, M 1446

Monteverde, M 397

Montgomery, S 217

Moreau, P 1660

Morel, F 685

Morgan, G 240

Morgan, MP 525

Morgillo, F 1648

Mori, M 947

Mori, M 1415

Morimura, R 740

Morita, H 1148

Morrison, R 1598

Mostafid, H 496

Motola-Kuba, D 1027

Motzer, RJ 646

Motzer, R 1475

Motzer, RJ 1587

Mross, K 1722

Muguruma, K 1668

Muhammad, A 909

Muir, M 1779

Muller, D 2016

Müller-Holzner, E 189

Müller, M 719

Muñoz, J 1367

Munster, PN 85

Muragaki, Y 1976

Murakami, S 1196

Muret, P 460

Murphy, MA 596

Murray, KE 1117

Murray, LJ 233

Musk, AW 1016

Muth, C 1262

Myers, RM 307

Myklebust, MP 756

Naase, M 1166

Nadayil, D 962

Nafees, S 1262

Nagai, T 1997

Nagai, Y 876

Nagasawa, A 1148

Nagase, H 1148

Nagata, H 740

Naguib, NNN 1274

Nagulapalli Venkata, KC 1833

Nagumo, Y 1779

Naik, R 1753

Nakagawa, K 763

Nakagawa M 405

Nakai, Y 1934

Nakajima, K 1415

Nakajima, K 947

Nakamoto, Y 1268

Nakamura, M 1205

Nakamura, S 290

Nakanishi, H 1196

Nakayama, N 666

Nakayama, S 133

Nakayama, S 792

Nam, SJ 923

Nandakumar, A 962

Narayan, R 490

Narita, M 1148

Narita, M 1148

Narod, S 1160

Nassanian, H 333

Natanson, KL 2016

Navarro, C 1866

Naya, Y 405

Naymark, M 909

Neal, RD 1

Neal, RD 1262

Neal, RD 1940

Negrier, S 1587

Negulescu, A-M 141

Neidle, S 14

Nelen, MR 2016

Nelson, CP 1854

Nennecke, A 1875

Nerich, V 460

Netto, GJ 414

Neuzillet, Y 1083

Neville, BA 1021

Newell, DR 1386

Niederacher, D 2016

Niedobitek, G 1980

Nie, F 1320

Nielsen, DL 678

Nielsen, FC 1526

Nieters, A 1866

Nie, Y 1486

Nigro, CL 1446

Nihlén, A 793

Niiranen, K 517

Nikitin, PA 429

Ning, Y 227

Ning, Y 1833

Nishida, N 1997

Nishimura, M 1953

Nishio, K 727

Nishio, K 763

Nishioka, N 1535

Niskanen, T 678

Nitsche, U 133

Nitsche, U 792

Nobbenhuis, MAE 817

Nodin, B 931

Nofech-Mozes, S 1160

Nohata, N 405

Nolte, S 970

Nomura, K 1205

Norat, T 1004

Nordenstedt, H 1011

Nordestgaard, BG 199

Noro, R 867

Norrild, B 1526

North, BV 1439

Nour-Eldin, NA 1274

Nunes, J 909

Nuriya, M 1807

Nyakas, M 1728

Nyhan, MJ 711

O'Brien, JP 1598

O'Brien, L 1697

O'Ceilleachair, A 805

Oda, I 727

Odedra, R 858

O'Donovan, TR 711

Ogawa, T 1196

Oh, E 923

Ohga, N 1214

Ohira, M 1668

Ohotski, J 1453

Ohtsu, A 666

Oikawa, K 1976

Oizumi, S 1953

Ojeda, B 1367

Okada, Y 1148

Okamoto, I 763

Okamoto, K 740

Okano, H 1148

Okano, HJ 1148

Okano, T 867

Okuyama, R 939

Olden, K 1535

Old, LJ 324

Olesen, F 1262

Oliveras-Ferraros, C 1406

Ollivier, L 854

Olmos, D 1379

Olsen, MH 1560

Olsen, NJ 1016

Olsson, A 1297

Omata, M 1934

O'Neill, S 1760

Oniscu, A 358

Onodera, Y 1214

Onteniente, S 460

Oosterwijk, E 916

Oosterwijk, JC 2016

Orange, C 383

Orange, C 702

Orange, C 1187

Orange, C 1453

Orešič, M 99

Ørntoft, TF 366

Orsulic, S 333

Ortendahl, JD 1571

Ortiz-Martínez, F 1367

Osako, T 1675

Osawa, T 1214

Osborn, D 1842

Osborn, M 1772

Osher, DJ 1460

Osler, M 988

Osorio, A 2016

Ostenfeld, MS 366

O'Sullivan, GC 711

Otsuji, E 740

Otto, N 348

Ouyang, Q 1512

Ouzzane, A 1083

Ovaska, K 517

Overvad, K 1004

Overvad, K 1866

Oya, M 290

Øyan, AM 1682

Ozaki, T 1976

Ozcelik, H 2016

Padath, T 490

Pagani, O 1618

Page, K 375

Pakola, S 678

Palaniappan, N 32

Paligo, M 2016

Palli, D 1004

Palli, D 1866

Pallis, A 1917

Palmieri, C 397

Pandha, HS 496

Pan, F 693

Panico, S 1866

Pankratz, VS 2016

Panneerselvam, A 1475

Paoletti, X 854

Papacharalbous, E 482

Paranjothy, S 1753

Paris, C 1346

Park, J 1095

Park, JO 1469

Park, PJ 733

Park, S 923

Park, SH 1469

Park, Y 596

Park, Y-H 923

Park, YS 1039

Park, YS 1469

Parson, W 189

Parwani, AV 2004

Pasche, B 307

Pasini, E 966

Paszat, L 1160

Patel, A 1753

Patel, D 1089

Patel, R 70

Patil, M 1466

Patil, S 1587

Paulin, F 955

Pawlowski, T 768

Payne, MJ 1466

Payne, RE 375

Payne, RE 1790

Paz-Ares, L 1379

Pchejetski, D 909

Peate, M 1053

Pecorelli, S 1543

Peel, DNY 1464

Peeters, M 1926

Peeters, PHM 1004

Peeters, PHM 1866

Peiró, G 1367

Peissel, B 2016

Pellat-Deceunynck, C 1660

Peltomäki, P 517

Pencavel, T 496

Pennison, MJ 307

Pennucci, C 658

Peock, S 2016

Perraki, M 1917

Perry, M 1095

Peschard, P 496

Pestereva, E 1702

Petasis, NA 1833

Peterlongo, P 2016

Peters, J 1766

Peters, TJ 1940

Peto, J 575

Peyrat, J-P 2016

Peyton, M 116

Pfisterer, J 633

Pharoah, PD 1798

Philip, PS 1166

Phillips, K-A 1053

Phillips, K-A 1618

Phillips, MA 490

Pichler, M 1826

Piedoux, S 460

Pietersma, D 1728

Pignot, G 1083

Pignot, G 1177

Pili-Floury, S 460

Pili, R 77

Pinato, DJ 1439

Pinney, SM 996

Pirie, K 210

Pisano, C 633

Pischon, T 1866

Pivot, X 460

Platte, R 2016

Plaxe, S 70

Pluschnig, U 25

Pluschnig, U 440

Polesel, J 222

Pollak, M 1335

Polterauer, S 1551

Polyzos, A 453

Ponzoni, M 966

Popov, P 1076

Poppe, B 1460

Popple, A 1306

Porta, C 1475

Porter, IWT 61

Possinger, K 274

Powell, N 45

Pradhan, PK 962

Prado, CMM 1583

Prehn, JHM 1499

Preisler-Adams, S 2016

Presul, E 189

Preusser, M 440

Price, KN 1618

Price, P 1766

Price, T 496

Print, C 1134

Proby, C 1446

Prochilo, T 658

Protheroe, AS 1466

Provenzano, E 1798

Pscherer, S 1123

Puistola, U 344

Pujade-Lauraine, E 1728

Pujade-Lauraine, E 633

Pujol, P 2016

Purdie, CA 397

Purdie, K 1446

Pusztai, L 1107

Pütter, C 1171

Pwint, T 468

Pyne, NJ 1453

Pyne, S 1453

Qayyum, T 1187

Qian, D 77

Qi, Y 1107

Quan, Y 1512

Queralt, B 1406

Quinlan, PR 397

Raban, N 633

Rabbani, ZN 916

Racine, A 1004

Racine, A 1866

Raeder, MB 1682

Rahhal, J 1551

Rahman, N 1234

Rainer, J 189

Rakovitch, E 1160

Ramage, JM 1306

Ramaker, R 307

Ramani, V 1689

Ramdass, S 525

Ramnath, T 962

Ramsey, S 279

Ranki, A 517

Ranson, M 468

Ran, ZH 1320

Rasmussen, F 1842

Ratner, E 1543

Ravaud, A 1475

Ray-Coquard, I 629

Rebbeck, T 2016

Recchia, F 658

Redaniel, MT 982

Redman, V 1431

Reeves, G 210

Régnard, J-F 1989

Reid, A 1016

Reid, JE 1095

Reinacher-Schick, A 1033

Reinthaller, A 1551

Rennert, G 2016

Ren, R 889

Rezai, K 673

Rhees, B 768

Ribi, K 1618

Riboli, E 1004

Richards, CH 2010

Richards, S 1556

Richly, H 1722

Rickwood, D 107

Rieck, G 45

Rijkaart, DC 975

Rijlaarsdam, MA 791

Rinaldi, S 1004

Ristamäki, R 428

Ritter, G 324

Roberts, JD 1535

Robertson, L 1234

Robey, IF 1280

Robinson, SP 1638

Robinson, SP 1960

Röcken, C 1033

Rodgers, SE 1481

Rod, NH 1560

Rodríguez-Blanco, J 1288

Rodríguez, C 1288

Rodríguez, L 1004

Rodríguez, L 1866

Rodriguez, R 1379

Roeder, I 1742

Rohan, TE 227

Rohrmann, S 1004

Rohrmann, S 1866

Rolland, P 1306

Romaguera, D 1866

Roodhart, JML 1901

Rookus, M 2016

Roque, D 1543

Rosenbaum, E 414

Rosenberg, DO 1833

Rosenberg, R 133

Rosenberg, R 792

Rosenquist, R 2016

Rose, P 1262

Rose, PW 1

Rose, PW 1940

Ross, G 1234

Ross, HJ 839

Rossig, C 1123

Rossing, M 1526

Ross, P 1766

Roswall, N 1004

Rothfuss, J 693

Roth, GA 904

Rothman, N 585

Rothman, N 1891

Roti, G 254

Rottenfusser, A 25

Rottenfusser, A 440

Roundhill, EA 1224

Rouprêt, M 1083

Rous, BA 1068

Rouzier, R 39

Roxburgh, CSD 2010

Royer, B 460

Rozendaal, L 975

Rozet, F 1083

Rozkrut, D 2016

Rubin, G 1262

Rubinsky, B 490

Rudas, M 25

Rudas, M 440

Rudek, MA 77

Ruijter, R 1728

Ruskeepää, A-L 99

Rustin, G 1766

Rutherford, MJ 1854

Rutherford, TJ 1543

Ryan, A 1910

Ryan, AJ 1960

Ryder, D 508

Ryoo, B-Y 1039

Ryu, M-H 1039

Ryu, M-H 1591

Saeb-Parsy, K 436

Safuwan, NAM 482

Saida, T 939

Saito, S 182

Sakai, K 763

Sakaizawa, K 939

Sakakibara-Konishi, J 1953

Sakurai, K 1668

Sakurai, T 1997

Salgia, R 839

Salmon, I 141

Salumbides, B 77

Salvesen, HB 1682

Samonigg, H 1826

Sampieri, K 1564

Sampo, M 1076

Sánchez, M-J 1866

Sánchez-Sánchez, AM 1288

Sánchez-Tejada, L 1367

Sandhu, S 1728

Sangar, V 1689

Santin, AD 1543

Sapkota, SD 962

Sasahira, N 1934

Sasaki, H 727

Sasaki, T 1934

Sasieni, P 1753

Sasieni, P 45

Saskin, R 1160

Sato, N 876

Sauer, L 909

Saunders, C 1053

Saunders, E 1697

Saunders, M 793

Saunders, NA 107

Saunders, VA 1772

Sautés-Fridman, C 1989

Savage, E 638

Savage, PM 1089

Savaraj, N 1481

Sawada, T 1668

Sawyer, E 1697

Sawyer, MB 1583

Scaggiante, B 166

Scardino, P 1095

Scartozzi, M 799

Schellens, JHM 1598

Scheulen, ME 1722

Schewnn, M 1891

Schierle, K 562

Schiltz, RL 116

Schlag, PM 1395

Schlegelberger, B 348

Schmelter, T 70

Schmid, J 1499

Schmid, P 447

Schmidt, S 189

Schmoor, C 1033

Schmutzler, R 2016

Schnyder, J 839

Schoen, RE 608

Scholz, A 348

Schöni-Affolter, F 447

Schreeder, MT 839

Schrevel, M 1520

Schultheis, B 1722

Schulz, P 348

Schussel, J 1314

Schuster, T 133

Schuster, T 792

Schwartz, PE 1543

Schwarz, S 719

Science, M 1626

Scott, IV 1306

Scott, JG 174

Scott, RJ 2016

Scott, S 1262

Scudiero, DA 1395

Seal, S 1234

Sebire, NJ 1089

Seckl, MJ 1089

Seddon, AM 883

Seebacher, V 1551

Segerström, L 1807

Seike, M 867

Seki, N 405

Seliger, B 1980

Selva-Nayagam, S 61

Seppä, K 1846

Serag, H 436

Serraino, D 222

Serraino, D 966

Serrano, P 2016

Servois, V 854

Sesboüé, R 426

Seth, R 496

Seynaeve, C 2016

Seywright, M 1187

Shahzad, N 222

Shanley, S 1234

Shao, C 889

Shapiro, J 61

Sharma, R 1439

Sharp, L 805

Shaw, H 1379

Shaw, JA 375

Shen, B 1486

Shenker, N 248

Shiang, CY 1107

Shibayama, M 133

Shibayama, M 792

Shikany, JM 227

Shimada, S 939

Shimada, Y 727

Shimokawa, T 867

Shindoh, M 1214

Shin, J-G 1591

Shinohara, N 1214

Shinozaki, H 1268

Shin, SJ 53

Shin, YK 923

Shin, YS 733

Shiozaki, A 740

Shoemaker, RH 1395

Short, D 1089

Shrestha, S 1583

Shuen, A 1460

Shu, XO 585

Sidon, L 775

Sidransky, D 1314

Siegel, F 1395

Sier, CFM 1495

Siersema, PD 1866

Siggberg, L 517

Siguero, M 1379

Silasi, D-A 1543

Silcocks, P 1259

Sillevis Smitt, PAE 538

Silverman, DT 1891

Simard, J 2016

Simes, J 1045

Simon, AE 1907

Simon, B 685

Simpson, GR 496

Sims, AH 1779

Singer, S 425

Sinha, R 1891

Sinha, R 608

Sinilnikova, OM 2016

Sipilä, L 517

Siracusano, S 166

Sisson, W 1481

Sivridis, E 846

Skarstein, A 756

Skjelbo Nielsen, MR 1866

Skjeldal, S 297

Skotte, L 1526

Slade, M 375

Sloane, R 508

Småstuen, MC 297

Smedby, KE 1850

Smeets, P 1926

Smeland, S 297

Smith, A 262

Smith, DP 638

Smith, E 1460

Smith, I 1760

Smith, J 1395

Smith, N 1766

Smith, PD 858

Smith-Warner, SA 1335

Snijders, PJF 817

Snijders, PJF 975

Snowdon, CF 1062

Snyder, SA 916

Soeno, C 867

Somogyi, AA 1772

Sondak, VK 85

Song, DY 1881

Song, S 1881

Song, S-X 1735

Song, Y 1881

Sørensen, M 678

Soto-Matos, A 1379

Soucy, P 2016

Souglakos, J 453

Soulie, M 1083

Spayne, J 1160

Specht, L 1526

Spendlove, I 1306

Spina, M 424

Spry, N 61

Srinivasan, V 262

Staines, A 805

Stålberg, K 1860

Stapley, S 1940

Stassen, J-M 678

Stearn, S 436

Stebbing, J 375

Stebbing, J 909

Steding-Jessen, M 988

Steele, N 793

Stefansson, IM 1682

Steger, GG 25

Steger, GG 440

Stegmaier, K 254

Steiner, MFC 1361

Steinert, F 562

Steinger, B 826

Stein, U 1395

Stenberg, Y 678

Stenmark-Askmalm, M 2016

Stephenson, JJ 839

Stevens, MCG 982

Stewart, GS 262

Stickles, XB 1967

Stiegler, C 719

Stiller, CA 1556

Stocken, DD 1431

Stockler, M 638

Stone, RA 2004

Stone, S 1095

Stoop, H 791

Stoppa-Lyonnet, D 2016

Stotz, M 1826

Straif, K 575

Strasak, AM 189

Stratigakou, V 1866

Stredder, C 70

Street, D 638

Stricker, P 638

Strickler, HD 227

Strumberg, D 1722

Stuart, M 1728

Sturge, J 909

Subramaniam, DS 839

Succop, P 996

Sugihara, K 1268

Sugimura, K 1415

Sugiyama, Y 126

Su, N 1790

Sundar, S 1464

Sundas, P 962

Sund, M 1004

Sund, M 1866

Sundström, J 428

Sung, JJY 1486

Sung, L 1626

Sutter, C 2016

Suzuki, A 1148

Suzuki, T 1148

Svane, D 1526

Sveinbjörnsson, B 1807

Svensson, T 1850

Svensson, T 1860

Swaisland, H 468

Syed, N 397

Syed, N 1446

Sylla, B 222

Symmans, WF 1107

Sy, S 1571

Szabo, C 2016

Szlosarek, P 1446

Tabernero, JM 1379

Tada, M 1934

Tagliabue, G 1004

Tait, B 1598

Takagane, A 1268

Takahashi, K 1268

Takahashi, T 126

Takahashi, T 947

Takahashi, Y 1268

Takakura, M 1205

Takanashi, M 1976

Takata, M 939

Takayama, T 126

Takeshita, H 740

Takezawa, K 763

Takiguchi, S 1415

Takiguchi, S 947

Takii, Y 1268

Takiuchi, H 1268

Takiuchi, H 666

Takubo, T 666

Talamini, R 1899

Talamini, R 222

Talamini, R 966

Talbot, DC 1466

Tamburini, J 1660

Tanaka, F 947

Tanaka, H 1196

Tanaka, H 1668

Tanaka, K 1415

Tanaka, M 1976

Tanaka, N 290

Tan, DS 1379

Tan, DS-W 1728

Tang, Y 1512

Taniguchi, H 727

Tanizaki, J 763

Tao, S 1424

Tappenden, P 805

Tarantini, L 585

Tarkkanen, M 1076

Taube, JM 1314

Tavora, FF 414

Tawadros, T 1689

Taylor, AM 262

Taylor, C 1160

Taylor, DG 14

Taylor-Harding, B 333

Taylor, M 1466

Taylor, SE 1581

Tejpar, S 1648

Teoh, N 1486

ter Haar, NT 1520

Teucher, B 1004

Teucher, B 1866

Therkildsen, MH 1526

Thewes, B 1053

Thielecke, L 1742

Thiery-Vuillemin, A 460

Thiruchelvam, D 1160

Tholander, B 1860

Thomas, S 1171

Thomassen, M 2016

Thompson, A 397

Thompson, A 955

Thompson, A 1446

Thompson, A 1618

Thompson, AM 1117

Thomsen, L 1134

Thürlimann, B 1618

Tibau, A 1367

Tibodeau, JD 314

Tijono, S 1134

Tikidzhieva, A 1239

Tilson, L 805

Timmermans, M 791

Timmons, R 148

Tin, AW 1153

Tirapo, C 2016

Tirelli, U 424

Tirelli, U 1899

Tischkowitz, MD 1460

Tiseo, M 658

Titze, U 1123

Tjønneland, A 1004

Tjønneland, A 1866

Toda, N 1934

Togawa, O 1934

Tomiak, E 1460

Tomita, N 1268

Tommasino, M 222

Tong, JL 1320

Tonin, P 1460

Touboul, E 39

Tourneur, L 1989

Townsend, PA 157

Trabert, B 1331

Tran-Thanh, D 1506

Travis, RC 1866

Tresca, P 673

Tresca, P 854

Trichopoulou, A 1004

Trichopoulou, A 1866

Trimbos, JBMZ 1520

Tristram, A 45

Troiani, T 1648

Troisi, R 1926

Trovik, J 1682

Trumpp, A 1742

Tsakmaki, V 846

Tsang, RY 6

Tsitlaidou, M 2016

Tsujino, T 1934

Tsuji, S 126

Tsujiura, M 740

Tsutsumi, R 666

Tudor, CS 1980

Tukiainen, E 1076

Tumino, R 1004

Tumino, R 1866

Tunariu, N 619

Turnbull, C 1234

Turner, N 1760

Tutt, A 1234

Tu, Z 693

Tymrakiewicz, M 1697

Tynelius, P 1842

Uchiyama, A 939

Ueno, N 1107

Uesaka, H 867

Ueshima, K 1997

Uhara, H 939

Uhlén, M 931

Uhlén, P 1807

Um, I 1779

Underhill, C 61

Underwood, MA 1187

Urbas, P 85

Usher, C 805

Vaccher, E 1899

Vacher, S 1177

Valeri, A 1083

Validire, P 1989

Valle, JW 508

Vamvakas, L 453

van Asperen, CJ 2016

Van Cutsem, E 1648

Van Damme, N 1926

van den Bent, MJ 538

van den Broek, CBM 1564

van den Eiden, LCG 269

van den Ouweland, AMW 2016

van der Avoort, IAM 269

van der Burg, SH 1520

van der Luijt, RB 2016

van der Reijden, JJ 1495

van de Velde, CJH 133

van de Velde, CJH 792

van de Velde, CJH 1564

Van de Wiele, C 1926

van Duijn, W 1495

Vangveravong, S 693

van Hazel, GA 61

van Herk, HADM 791

van Herk-Sukel, MPP 1564

van Kemenade, FJ 817

van Kemenade, FJ 975

van Kempen, LCLT 269

van Leenders, GJLH 791

Vanlemmens, L 673

Van Maerken, T 141

van Rijswijk, E 1262

Varghese, DS 116

Varon-Mateeva, R 2016

Vazquez-Martin, A 1406

Vecchiarelli, J 1506

Vecchione, L 1648

Vedder, JEM 269

Vedsted, P 1262

Venat-Bouvet, L 2016

Vennin, P 2016

Vergote, I 629

Vergote, I 633

Verheijen, RHM 975

Vermaat, J 284

Verma, Y 962

Verry, H 1045

Verschraegen, C 70

Verset, L 141

Verspaget, HW 1495

Verweij, W 975

Vessella, RL 768

Vieillefond, A 1177

Villadsen, SB 366

Villanueva-Rodríguez, G 1027

Vineis, P 1004

Vineis, P 1866

Viney, R 638

Visakorpi, T 768

Vitagliano, D 1648

Vivenza, D 397

Voest, E 284

Voest, EE 1901

Vogl, TJ 1274

Volgger, B 633

Volkmer, B 970

von Knebel Doeberitz, M 1239

von Wachenfeldt, A 2016

Vujaskovic, Z 916

Vuoristo, A 99

Wakelam, MJ 1431

Waldert, M 904

Waldmann, A 970

Walker, G 1481

Walker-Samuel, S 1638

Walker-Samuel, S 1960

Walker, T 157

Wallis, MG 1611

Walraven, M 284

Walsh, C 805

Walsh, CS 333

Walter, FM 1262

Walter, FM 1940

Walther, W 1395

Wandeler, G 447

Wanders, J 1598

Wang, B 1107

Wangefjord, S 931

Wang, F 1790

Wang, H 1446

Wang, J 1605

Wang, J 1772

Wang, J-P 1735

Wang, L 1134

Wang, L 1735

Wang, L-CS 1134

Wangpaichitr, M 1481

Wang, S 1605

Wang, W 1512

Wang, X 2016

Wang, Y 70

Wang, Y 1512

Wan, X-B 1735

Wappenschmidt, B 2016

Ward, B 375

Ward, J 638

Wardle, J 1907

Ward, MH 1891

Ward, ST 1431

Ward, T 1766

Ward, VK 92

Wareham, N 1866

Wareham, NJ 1004

Warren-Perry, M 1234

Watanabe, M 182

Watanabe, M 876

Watanabe, M 1268

Watanabe, T 1268

Watanabe, T 1997

Waterton, JC 1960

Watson, EK 1

Waxman, J 909

Weber, JS 85

Weber, S 1171

Webster, RM 32

Wehle, D 414

Weinstock, MA 970

Weissfeld, JL 2004

Weller, D 1

Weller, D 1262

Wenham, RM 1967

Wen, S 1374

Wentzensen, N 596

Werner, HMJ 1682

Westra, WH 1314

Wetzig, N 1045

Whitaker, KL 1907

White, DL 1772

Whitley, E 1842

Whyte, S 805

Widschwendter, M 189

Widschwendter, M 1910

Wiedenmann, B 348

Wik, E 1682

Wiklund, ED 366

Wild, P 1346

Wilkinson, C 1

Wilkinson, R 1697

Wilkinson, RW 858

Willett, WC 1335

Willian, M 148

Wilson, S 1431

Wimberger, P 629

Winkler, S 1134

Win, SJ 92

Winstedt, L 678

Winter, E 1826

Witteveen, E 284

Wolk, A 592

Wolk, A 603

Woll, PJ 1153

Wones, R 996

Wong, AJ 883

Wooley, K 107

Wratten, C 61

Wright, K 436

Wu, B-W 324

Wu, CJ 1481

Wu, C-W 1486

Wu, J 1512

Wu, JW 1891

Wu, K 1335

Wu, L 1512

Wullschleger, S 1117

Wu, P-H 1735

Wyatt, JC 826

Xiao, SD 1320

Xie, Y 889

Xiong, Y 1967

Xu, C 1506

Xu, Q 1320

Xu, XT 1320

Xu, Y 553

Xynogalos, S 453

Xyrafas, A 1917

Yaghjyan, L 996

Yagi, K 126

Yagüe, E 1790

Yagüe, E 375

Yamada, Y 727

Yamaguchi, H 763

Yamamizu, K 1148

Yamamoto, K 1214

Yamasaki, M 947

Yamasaki, M 1415

Yamashita, JK 1148

Yanagihara, K 727

Yang, CT 307

Yang, D 1833

Yang, G 1512

Yang, G 585

Yang, H-K 733

Yang, W-M 1248

Yang, Z-L 1735

Yap, TA 1379

Yashiro, M 1535

Yashiro, M 1668

Yasui, M 1807

Yates, DR 1083

Yeh, T-K 475

Yen, K-J 475

Yen, S-C 475

Yeoh, K-W 651

Ye, S 889

Ye, W 1004

Ye, W 1866

Yi, JH 1469

Yim, D-S 1469

Yi, N 307

Yoon, DH 1039

Yoshida, K 126

Yoshida, M 666

Yoshii, M 1668

Yoshimura, A 867

Yoshino, H 405

You, M 1481

Young, RJ 1153

Young, SL 92

Younus, A 1095

Yuan, D 889

Yu, J 1486

Yu, K 585

Yu, KJ 206

Zaal, A 817

Zabaglo, L 1760

Zaidi, SAA 375

Zambito, F 748

Zanconati, F 166

Zanelli, F 658

Zangos, S 1274

Zebrowski, A 1790

Zeestraten, ECM 133

Zeestraten, ECM 792

Zeitler, K 719

Zeng, C 693

Zenk, J 719

Zennaro, C 166

Zerbib, M 1177

Zeschnigk, M 1171

Zhang, G 1248

Zhang, J 693

Zhang, R-W 1248

Zhang, X 1335

Zhang, Z 414

Zhao, G 1486

Zhao, M 77

Zhao, Y 1605

Zha, X-m 1605

Zheng, W 585

Zhu, F 748

Zhu, MM 1320

Zielinski, CC 25

Zielinski, CC 440

Zimmerman, JW 307

Ziras, N 453

Zlobec, I 1713

Zweemer, AJM 1779

Zweifel, M 1766

Zwiebel, J 77

